# *Retracted: *Experimentally investigated the asparagus (*Asparagus officinalis* L.) drying with flat‐plate collector under the natural convection indirect solar dryer

**DOI:** 10.1002/fsn3.603

**Published:** 2018-02-21

**Authors:** Fahim Ullah, Min Kang, Mansoor Khan Khattak, Said Wahab

**Affiliations:** ^1^ College of Engineering Nanjing Agricultural University Nanjing China; ^2^ Guanyun Research Institute for Modern Agricultural Equipment Nanjing Agricultural University Guanyun China; ^3^ Department of Agricultural Mechanization FCPS The University of Agriculture Peshawar Peshawar Pakistan; ^4^ Department of Food Science and Technology FNS The University of Agriculture Peshawar Peshawar Pakistan

## Abstract

The above article, published online on 21 February 2018 in Wiley Online Library (wileyonlinelibrary.com), has been retracted by agreement between the authors, the journal Editor in Chief, Y. Martin Lo, and Wiley Periodicals, Inc. The retraction has been agreed at the authors’ request due to unattributed overlap between this article and the following article published in the *Journal of Mechanical Engineering and Sciences*, “Analysis of ginger drying inside a natural convection indirect solar dryer: An experimental study” by S. K. Sansaniwal and M. Kumar, Volume 9, pp. 1671–1685.

Fahim Ullah[1] | Min Kang[1,2] | Mansoor Khan Khattak[3] | Said Wahab[4]

RETRACTED

## Abstract

In this study, a natural convection indirect solar cabinet dryer has been fabricated to study the drying behavior of Asparagus (*Asparagus officinalis* L.) in terms of its convective heat transfer coefficient and moisture removing rate (% db). The experiments were conducted during the months of June, July, and August 2016, at College of Engineering, Nanjing Agricultural University, Nanjing (32°3′36.92″ N and 118°47′48.76″ E), China. Linear regression method was applied to the experimental data to evaluate the Nusselt number constant. From the results of the experiments, it was observed that convective heat transfer coefficient decreased with the increasing mass of the samples. Similarly, the progression of drying months with variation from 0.59 to 5.42 W/m$^2$ °C for the different mass of samples was noted. Therefore, from the results of the experiment, it was reported that moisture removing rate increased with the increase in mass of asparagus samples and significantly decreased with progression of drying months. Similarly, during experiments, the average collector efficiency was noted to vary from 14.97% to 16.14% under the increasing and decreasing trends of solar irradiations from morning to noon and noon to evening, respectively. For describing the drying behavior of the different mass of Asparagus samples, modified Henderson and Pabis were reported. During experiments, experimental error in terms of percent uncertainty was observed in the range from 29.19% to 46.25%.

Asparagus, convective heat transfer coefficient, flat-plate collector, indirect solar dryer, moisture, natural convection

## NOMENCLATURE

| Symbol | Meaning | Unit | Symbol | Meaning | Unit |
|---|---|---|---|---|---|
| $M_{initial}$ | Initial moisture removing rate | %, dry basis | $Q_o$ | Total heat output | J/sec |
| $Mo$ | Initial moisture content | | $V_o$ | Average air velocity at collector outlet | m/s |
| $Me$ | Equilibrium moisture content | | $A_{oc}$ | Area of collector outlet | m$^2$ |

| Symbol | Meaning | Unit | Symbol | Meaning | Unit |
|---|---|---|---|---|---|
| $W_w$ | Weight of wet Asparagus | g | $\rho v$ | The density of air | kg/m$^3$ |
| $W_d$ | The weight of dry Asparagus | g | $T_{oc}$ | the temperature at collector outlet | ˚C |
| $M_{exp,i}$ | Experimental moisture ration | % | $T_{ic}$ | Temperature at collector inlet | ˚C |
| $M_{pre,i}$ | Predicated moisture ration | % | $T_c$ | Product temperature | ˚C |
| δ | The standard deviation | – | $T_e$ | Product surrounding temperature | ˚C |
| *No* | Number of sets | | *cv* | Specific heat of air | J/kg˚C |
| *N* | Number of observations | | $Q_i$ | Heat input | J/sec |
| *n* | Number of drying model constant | | *I* | Solar irradiation | W/m$^2$ |
| *Nu* | Nusselt number | | $A_c$ | Apparent area of the collector | m$^2$ |
| *Re* | Reynolds Number | | *X* | Characteristic length | |
| *Gr* | Grashof Number | | *P(T)* | Vapor pressure at temperature | T/N/m$^2$ |
| *Pr* | Prandtl Number | | $\mu_v$ | Dynamic viscosity of air | kg/m.s |
| *C and n* | Constant term | | *Kv* | Thermal conductivity | W/m.˚C |
| *hc* | The convective heat transfer coefficient | W/m$^2$°C | $R^2$ | Coefficient of determination | – |
| $X^2$ | Chi-square | – | RMSE | Root-mean-square error | – |

RETRACTED

## 1 | INTRODUCTION

Asparagus (*Asparagus officinalis* L.) is an herbaceous, perennial plant, growing to 100–150 cm tall, with stout stems with much-branched, soft vegetation which was commonly grown in temperate climate worldwide (Azharul Karim & Hawlader, 2005). The basic ingredients of Asparagus were energy, starch, proteins, mineral matters, vitamins, fats, and carbohydrates. Asparagus is an important ingredient of the food with high nutritional value and has become a compulsory item in the kitchen (Bhagat & Lawankar, 2012). It is not only used to add food palatability, but it is also widely used in medicines, bakery products, wine and meat products, a toiletry product, etc. (Denis & Mikulic-Petkovsek, 2017). Asparagus is the most important cash crop of the world, cultivated in China, Pakistan, Indian, Afghanistan, Uzbekistan, Japan, and Indonesia. The china is the largest producer of Asparagus, contributing about 45.48% of the total world's Asparagus production with a total production of 17 million tons a year (Deshmukh, Varma, Yoo, & Wasewar, 2013). About half of the total production of Asparagus is being consumed as white and red Asparagus, whereas the remaining 30% is converted into dry Asparagus for medicinal purposes, and 20% is used as seed material (Deshmukh, Varma, Yoo, & Wasewar, 2014).

Agricultural product drying has a vital role in the preservation and shelf life improvement of the product after harvesting (Eze & Agbo, 2011). In developing countries, sun-drying is a popular, effective, and economical method for drying of food and herbal products. Sun-drying is a common food preservation technique used to control the moisture content of the agricultural products (Gürlek, Özbalta, & Güngör, 2009). Traditionally, herbs like Asparagus dried in open sun are very much dependent on the availability of sunshine, require large drying space and long drying time (Hoque, Bala, Hossain, & Uddin, 2013). In order to fulfill the quality food product requirement of the growing population, efficient and affordable drying methods should be practiced. Today's world of growing technology has facilitated various types of drying systems which prevent the deterioration of products along with reduced product drying time. But these drying technologies are not economically feasible as they involve high capital investment and energy cost (Inci & Dursun, 2004; Jayashree, Visvanathan, & Zachariah, 2014).

In the present time of emerging solar energy applications, solar drying is one of the most promising alternatives to sun-drying. It is an ecofriendly and economically viable technology, thus being used in most developing countries (Karna & Koo, 2017). Different researchers have studied the using of various categories of solar dryers for the drying characteristics of different vegetables and fruits. According to Karna & Koo, 2017; Khoukhi & Maruyama, 2006; Kong, Lin, & Li, 2013; they reported the 26.25 W/m$^2$ for convective heat transfer coefficient of asparagus sample under the sun-drying condition. Other studies (Kumar, Khatak, Sahdev, & Prakash, 2011; Kumar, Sansaniwal, & Khatak, 2015; Kumar & Tiwari, 2009) have been reported that drying rate under the hybrid dryer was greater than sun-drying with the efficiency of 15% during the summer season. Modified Henderson and Pabis were reported to be best suited to describe the drying behavior of Asparagus (Lu, Yu, & Ding, 2003). The drying characteristics of Asparagus undertray and heat pump-assisted dehumidified drying were also incorporated by single and two stages drying, which reduced the drying time by 59.32% at 40°C (Kumar, 2013a,b). Peeled and unpeeled Asparagus drying under sun-drying and solar cabinet dryer have been compared, and better drying rate was observed in solar drying against sun-drying (Hoque et al., 2013). Other researchers (Kumar, 2013a, 2014; Norm, 2003) have been reported the drying behavior of asparagus at four different drying air temperatures with the fixed air velocity of 1.3 m/s. They concluded that moisture content from 87 to 6% was observed to be reduced on a wet basis.

Drying characteristics of Asparagus having a slicing of different lengths varying from 5 to 50 mm were studied using different drying methods like sun-drying, solar tunnel drying, and cabinet tray drying (Maskan, Kaya, & Maskan, 2002). It was also observed that the

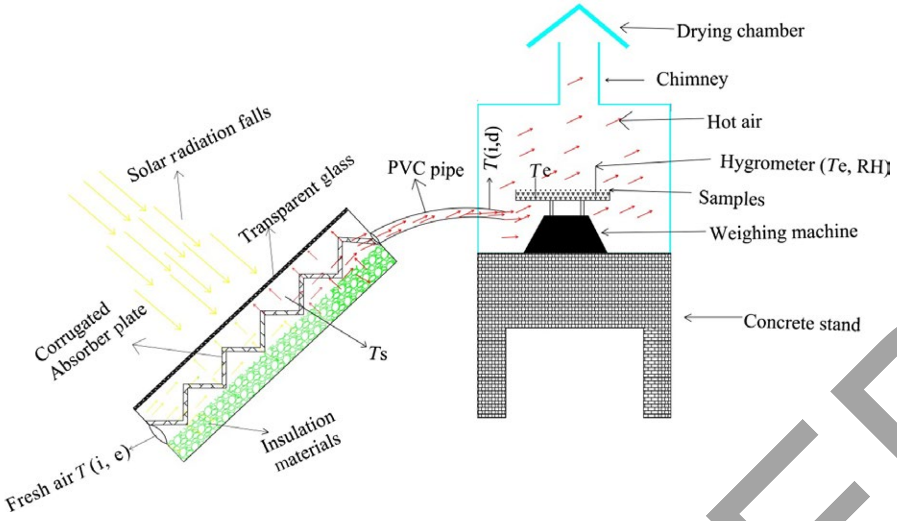

**FIGURE 1** The schematic view of flat-plate collector under the natural convection indirect solar dryer; *T*(i.e.) is the fresh air inlet temperature in (°C), PVC means poly vinyl chloride pipe, $T_s$ is the absorber surface temperature in (°C), *T*(*i, d*) is the inlet pipe diameter temperature in (°C), $T_e$ is the product surrounding temperature in (°C), $T_c$ is the product temperature in (°C), and RH is the relative humidity of the product in (%)

drying of whole Asparagus shrubs under open sun took maximum time, followed by solar tunnel drying (Ghaffar, 1995; Neiton, Claudio, & Marcos, 2017; Phoungchandang & Saentaweesuk, 2011). A photovoltaic powered indirect forced convection solar dryer was developed for drying Asparagus with maximum collector temperature of 66 and 81°C without and with the use of reflector mirror, respectively. Drying kinetics of Asparagus shrubs under blanched and nonblanched conditions was presented using the hybrid solar dryer, and the drying rate dependency on product shape, size, and drying air temperature was observed. Drying air temperature of 70°C was reported best for better quality drying of Asparagus shrub. Modified Henderson and Pabis were reported best to describe the drying characteristics of Asparagus shrub (Tiago & Maria, 2014; Tiwari, Tiwari, & Al-Helal, 2016). Thin layer solar drying of Asparagus was carried out for different mass flow rates of 0.06 and 0.12 kg/s with an average temperature of 54 and 44°C, respectively, for which Modified Henderson and Pabis were reported to be most appropriate to describe the drying behavior of Asparagus (Lu et al., 2003; Rahman, Karuppaiyan, Kishore, & Denzongpa, 2009; Tesfamariam, Kahsay, Kahsay, & Hagos, 2015).

A solar dryer was designed by the researchers (Xiong, Yuting, & Chongfang, 2012; Yuezhao & Jiang, 2007) with evacuated tube collectors for Asparagus drying at different air mass flow rates in the range of 4–5 m/s and reduced the moisture content of the product from 85.62% to 0.92%. The drier efficiency was reported to vary from 31 to 40.4% for different air mass flow rates. Overall, the dryer was suggested to be better than other dryers in terms of quality and drying rate (Tiago & Maria, 2014; Tiwari et al., 2016). Drying characteristics of Asparagus using a mixed mode solar cabinet dryer were investigated by reducing its moisture from 621.50 to 12.19% (Kalogirou, 2009; Meng, Yuezhao, & Yang, 2013). Other studies (Fahim, Mansoor, Maazullah, Lubna, & Kamran, 2016; Gang, Huide, & Jie, 2010; Shanmugasundaram & Janarthanan, 2013) have been reported that solar dryer to be better than a sun-drying method for asparagus samples drying in the aspects of quality. In this study, an indirect natural convection solar dryer has been fabricated to study the drying kinetics of Asparagus shrubs in the meteorological conditions of Nanjing, China. Furthermore, solar flat-plate collector efficiency has also been evaluated for the given drying time interval.

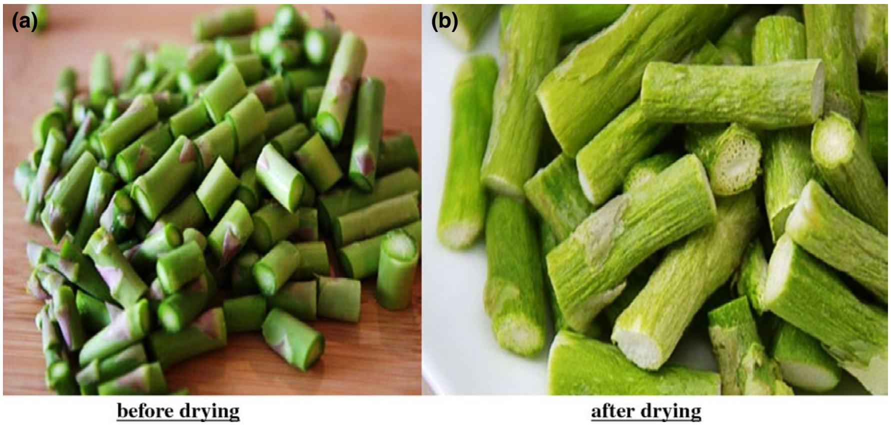

**FIGURE 2** Asparagus samples before drying (a) and after drying (b)

RETRACTED

**TABLE 1** Thin layer of drying models

| S. No | Model Name | Model | Cited |
|---|---|---|---|
| 1 | Wang and Singh | $MR = 1 + at + kt^2$ | Togrul & Pehlivan (2003) |
| 2 | Henderson & Pabis | $MR = a \exp(-kt)$ | Tesfamariam et al. (2015) |
| 3 | Modified Henderson and Pabis | $MR = a \exp(-kt) + b \exp(-gt) + c \exp(-ht)$ | Neiton et al. (2017) |
| 4 | Logarithmic | $MR = a \exp(-kt) + c$ | Karna & Koo (2017) |
| 5 | Newton | $MR = \exp(-kt)$ | Denis & Mikulic-Petkovsek (2017), Inci & Dursun (2003) |

## 2 | MATERIALS AND METHODS

### 2.1 | Description of experimental setup

The flat-plate collector was fabricated for the purposes of asparagus drying under the natural convection indirect solar dryer in the College of Engineering, Nanjing Agricultural University, Nanjing, China. The design of the solar dryer was installed at the latitude (32°3′36.92″ N) and longitude (118°47′48.76″ E) of the experimental area for getting maximum solar irradiance. The solar dryer was composed of two basic elements, *that is*, flat-plate collector and drying chamber with the dimensions of 1.3 × 1 m and 0.39 × 0.43 × 0.51 m, respectively. The flat-plate collector was consisting of a black-coated aluminum sheet, insulation material (air heating), and a transparent glass sheet (8 mm), which were used for getting the maximum efficiency. Similarly, drying chamber was fabricated from a wood material and fully insulated with insulation material to minimize the heat losses. It was connected with collector through PVC pipe which was used for allowing the heated air from collector to dry chamber. The 78% initial moisture content of fresh asparagus samples were put on the wire mesh tray for drying under natural convection process. After 1-h time interval, the electronic weighing balance (Model TJ-6000) was used for the determination of weight reduction of the product with least count of 0.1 gm with the capacity of 6 kg. For the determination of relative humidity and surrounding temperature of the product, digital hygrometer (Model HT-315) was placed above the product surface, and the inlet and outlet air temperatures were measured with thermocouples (Model PT-100) with the accuracy of ± 0.1°C. Therefore, solar power meter (Model, WACO-206) was used for the measuring of solar irradiance during the experiment. The schematic view of natural convection indirect solar dryer is shown in Figure 1.

### 2.2 | Description of experimental procedure

For experimental work, we took fresh asparagus samples with the numbers of 78 and 48 from the available local market in Nanjing, China, and washed with distilled water. The samples were cut cylindrically with the length and diameter of 3.0 cm and 1.7 cm, respectively, and put in the tray placed on weighing balance. The data were recorded in the month of June, July, and August 2016, within a time interval of 9:00 AM to 6:00 PM. The different numbers of asparagus samples were put in different trays and placed on the digital electronic balance for the determination of moisture content removal within each hour drying. Dryer inlet temperature, product surface temperature, inlet and an outlet temperature of the drying chamber, and absorber plate temperature were measured with the using of thermocouples. The experimental observation data were recorded after each hour of drying, and the drying was discontinued the constant weight of the samples were achieved. The difference in weight directly gave the quantity of water content evaporated during any time interval. Wet and dried Asparagus samples are shown in Figure 2.

The data obtained from the measurements of Asparagus weight were used for drying kinetics analysis of Asparagus in terms of

**TABLE 2** Statistical parameters obtained from selected thin layer models for solar cabinet drying Asparagus; $R^2$ is the coefficient of determination, $X^2$ is the reduced chi-square, and RMSE is the root-mean-square error

| | Asparagus samples (78) | | | Asparagus samples (48) | | |
|---|---|---|---|---|---|---|
| Model name | $R^2$ | RMSE | $X^2$ | $R^2$ | RMSE | $X^2$ |
| Wang & Singh | 0.873 | 0.120 | 1.843 | 0.845 | 0.154 | 2.243 |
| Henderson and Pabis | 0.761 | 0.238 | 4.937 | 0.736 | 0.263 | 5.486 |
| **Modified Henderson and Pabis** | **0.997** | **0.038** | **0.016** | **0.979** | **0.028** | **0.138** |
| Logarithmic | 0.817 | 0.179 | 3.39 | 0.790 | 0.208 | 3.864 |
| Newton | 0.943 | 0.071 | 0.502 | 0.907 | 0.101 | 0.945 |

RETRACTED

RETRACTED

**TABLE 3** Experimental data during natural convection indirect solar drying of different no. of Asparagus samples; $T_s$ is the absorber surface temperature in (°C), $T_{(i,c)}$ is the temperature at collector inlet in (°C), $T_{(o,c)}$ is the temperature at collector outlet in (°C), $T_c$ is the product temperature in (°C), $T_e$ is the product surrounding temperature in (°C), $M_{evp}$ is the moisture evaporation in (g), and $M_{removing\ rat}$ is the moisture removing rat in the products with the unit of (%db)

| Time (h) | $T_s$ (°C) | $T_{i,c}$ (°C) | $T_{o,c}$ (°C) | $T_c$ (°C) | $T_e$ (°C) | $M_{evp}$ (g) | $M_{removing\ rat}$ (%db) |
|---|---|---|---|---|---|---|---|
| 78 no. of Asparagus samples in the month of June 2016 | | | | | | | |
| 9.00 | 48.2 | 25.8 | 43.7 | 26.5 | 26.5 | – | – |
| 10.00 | 59.6 | 31.2 | 57.4 | 35 | 34.3 | 8.6 | 1.6 |
| 11.00 | 65.8 | 32.8 | 64.3 | 38.5 | 38 | 17 | 3.4 |
| 12.00 | 70.1 | 34 | 68.8 | 40.9 | 40.4 | 26.1 | 5.7 |
| 1.00 | 69.9 | 36 | 69.8 | 42.3 | 41.8 | 27.4 | 6.4 |
| 14.00 | 69.2 | 36.1 | 68.2 | 42 | 41.9 | 25 | 6.2 |
| 15.00 | 61.1 | 35.6 | 62.7 | 41 | 40.8 | 23 | 6.0 |
| 16.00 | 53.2 | 33.2 | 54.6 | 38.3 | 38.1 | 22.2 | 6.3 |
| 17.00 | 42.7 | 32.4 | 43.3 | 33.8 | 33.4 | 16 | 4.5 |
| 18.00 | 40.2 | 30.7 | 41.6 | 31.9 | 32.2 | 14.7 | 4.2 |
| Month of July 2016 | | | | | | | |
| 9.00 | 34.4 | 23.2 | 29.1 | 22.5 | 21.9 | – | – |
| 10.00 | 52.8 | 28.6 | 49 | 30.5 | 29.5 | 18.1 | 5.5 |
| 11.00 | 62.3 | 30.7 | 60.6 | 36.6 | 35.7 | 13.7 | 4.3 |
| 12.00 | 69.5 | 33.2 | 67.1 | 39.8 | 38.9 | 15.9 | 5.2 |
| 1.00 | 71.7 | 35.2 | 69 | 41.8 | 40.9 | 14.4 | 5.0 |
| 14.00 | 70.5 | 36.1 | 68.6 | 42.2 | 41.6 | 15.9 | 5.8 |
| 15.00 | 64.6 | 35.5 | 64.7 | 41.9 | 41.3 | 13.6 | 5.2 |
| 16.00 | 57 | 33.3 | 57 | 39.1 | 38.6 | 11.2 | 4.4 |
| 17.00 | 46.4 | 32.7 | 45.8 | 35.4 | 34.9 | 8.1 | 3.4 |
| 18.00 | 35 | 31 | 34 | 30 | 30 | 9.7 | 4.3 |
| Month of August 2016 | | | | | | | |
| 9.00 | 36.40 | 24.00 | 32.30 | 24.10 | 23.90 | – | – |
| 10.00 | 49.80 | 28.50 | 46.60 | 31.50 | 30.50 | 11.40 | 5.2 |
| 11.00 | 56.90 | 32.40 | 53.40 | 36.50 | 35.20 | 10.50 | 4.8 |
| 12.00 | 77.30 | 34.00 | 69.90 | 43.40 | 42.10 | 11.70 | 5.7 |
| 13.00 | 70.20 | 33.20 | 64.90 | 44.80 | 43.20 | 11.90 | 6.4 |
| 14.00 | 68.20 | 36.10 | 61.40 | 42.20 | 40.90 | 7.20 | 3.8 |
| 15.00 | 70.60 | 38.00 | 66.70 | 46.20 | 44.70 | 7.60 | 4.3 |
| 16.00 | 67.40 | 372.00 | 62.60 | 45.50 | 44.20 | 6.80 | 4.0 |
| 17.00 | 52.30 | 36.60 | 50.10 | 40.30 | 39.20 | 4.50 | 2.9 |
| 18.00 | 36.10 | 33.20 | 35.80 | 32.60 | 31.50 | 3.40 | 2.0 |
| 48 no. of Asparagus samples in the month of June 2016 | | | | | | | |
| 9.00 | 36.40 | 23.60 | 30.80 | 24.90 | 25.34 | – | – |
| 10.00 | 51.90 | 36.70 | 49.30 | 31.95 | 31.63 | 9.00 | 3.1 |
| 11.00 | 63.20 | 31.60 | 57.10 | 34.65 | 35.84 | 14.10 | 5 |
| 12.00 | 68.30 | 32.80 | 65.70 | 42.60 | 44.31 | 17.20 | 5.5 |
| 13.00 | 71.90 | 34.80 | 68.20 | 42.60 | 44.31 | 17.20 | 6.6 |
| 14.00 | 72.00 | 35.70 | 67.90 | 43.35 | 45.16 | 15.40 | 6.3 |
| 15.00 | 69.30 | 36.30 | 65.50 | 43.70 | 45.52 | 17.40 | 7.9 |
| 16.00 | 61.50 | 35.60 | 59.10 | 42.25 | 43.90 | 16.00 | 7.9 |

(Continues)

RETRACTED

**TABLE 3** (Continued)

| Time (h) | $T_s$ (°C) | $T_{i,c}$ (°C) | $T_{o,c}$ (°C) | $T_c$ (°C) | $T_e$ (°C) | $M_{evp}$ (g) | $M_{removing\ rat}$ (%db) |
|---|---|---|---|---|---|---|---|
| 17.00 | 49.10 | 33.10 | 48.60 | 37.85 | 39.21 | 11.80 | 6 |
| 18.00 | 41.00 | 31.50 | 38.80 | 34.70 | 35.52 | 7.90 | 4.2 |
| Month of July 2016 | | | | | | | |
| 9.00 | 37.60 | 28.10 | 34.70 | 27.20 | 27.91 | – | – |
| 10.00 | 55.00 | 31.10 | 52.80 | 33.95 | 34.13 | 15.00 | 8.6 |
| 11.00 | 68.30 | 33.10 | 65.00 | 39.55 | 40.50 | 10.80 | 6.4 |
| 12.00 | 74.90 | 38.40 | 71.00 | 43.20 | 43.85 | 12.80 | 7.9 |
| 13.00 | 72.60 | 38.80 | 72.00 | 45.10 | 45.30 | 17.50 | 13 |
| 14.00 | 70.60 | 39.20 | 70.90 | 46.15 | 45.88 | 13.70 | 11.6 |
| 15.00 | 64.30 | 38.50 | 66.10 | 45.55 | 45.11 | 13.80 | 12.9 |
| 16.00 | 57.50 | 37.50 | 58.40 | 42.80 | 42.49 | 8.40 | 8.3 |
| 17.00 | 47.40 | 35.20 | 49.00 | 39.60 | 39.29 | 4.80 | 4.5 |
| 18.00 | 37.50 | 34.70 | 37.40 | 34.70 | 34.65 | 3.30 | 3.7 |
| Month of August 2016 | | | | | | | |
| 9.00 | 37.30 | 28.00 | 33.40 | 27.20 | 27.30 | – | – |
| 10.00 | 51.20 | 31.40 | 49.80 | 34.50 | 34.27 | 5.50 | 6 |
| 11.00 | 56.70 | 32.50 | 58.80 | 40.70 | 39.98 | 7.00 | 8.1 |
| 12.00 | 66.50 | 36.50 | 97.50 | 45.50 | 44.54 | 6.30 | 7.6 |
| 13.00 | 73.20 | 39.00 | 72.20 | 50.10 | 48.03 | 3.60 | 4.5 |
| 14.00 | 59.30 | 37.40 | 58.30 | 45.20 | 44.21 | 4.50 | 6.1 |
| 15.00 | 65.10 | 38.80 | 64.10 | 47.30 | 46.20 | 1.00 | 1.9 |
| 16.00 | 59.80 | 38.00 | 60.20 | 46.50 | 45.48 | 1.00 | 1.9 |
| 17.00 | 49.90 | 37.30 | 50.20 | 42.50 | 41.61 | 0.60 | 1.6 |
| 18.00 | 39.30 | 36.80 | 39.30 | 36.30 | 36.70 | 0.30 | 1.4 |

moisture removing rate. The moisture removing rate was expressed on a dry basis. The experimental data were fitted to the already published thin layer drying models using nonlinear regression analysis as shown in Table 1. Equation (1) was used for the determination of moisture removing rate of the product. The moisture ratio of Asparagus during the drying can be obtained from equation (2).

$$M_{\text{initial}} = \frac{W_w - W_d}{W_d} \times 100 \tag{1}$$

$$\text{MR} = \frac{M - M_e}{M_o - M_e} \tag{2}$$

From the literature, it was observed that the Asparagus should be dried from its average initial moisture content of 89% to the final moisture content of 8% (Fahim, Mansoor, et al., 2016; Xiong et al., 2012). For the determination of the suitability of best thin layer drying model, the following $R^2$, $X^2$, and RMSE were considered to be the primary criteria as given in Equations (3) and (4).

$$X^2 = \frac{\sum_{i=1}^{N} (\text{MR}_{\exp,i} - \text{MR}_{\text{pre},i})^2}{N - n} \tag{3}$$

$$\text{RMSE} = \frac{1}{N} \left[ \frac{\sum_{i=1}^{N} (\text{MR}_{\exp,i} - \text{MR}_{\text{pre},i})^2}{N} \right]^{1/2} \tag{4}$$

The model suitability was determined by considering the higher value of the coefficient of determination and least value of chi-square and root-mean-square error (Phoungchandang & Saentaweesuk, 2011; Rahman et al., 2009). Statistical parameters obtained from selected thin layer drying models are given in Table 2. It can be observed that the modified Henderson and Pabis have the highest value of the coefficient of determination $R^2$ and corresponding least value of chi-square ($X^2$) and root-mean-square error (RMSE) among the five models used. So it has been concluded that the modified Henderson and Pabis are best suited for describing the drying behavior of different Asparagus mass samples.

## 2.3 | Theoretical considerations

The convective heat transfer coefficient for evaporation was determined using the following relations (Neiton et al., 2017; Yuezhao & Jiang, 2007). Equation 6 represents the rate of heat utilized to evaporate moisture. While substituting $h_c$ from equations (5) and (6) becomes equation (7). The moisture evaporated is determined by dividing equation 7 by latent heat of vaporization ($\lambda$) and multiplying the area of the tray ($A_t$) and drying time interval ($t$).

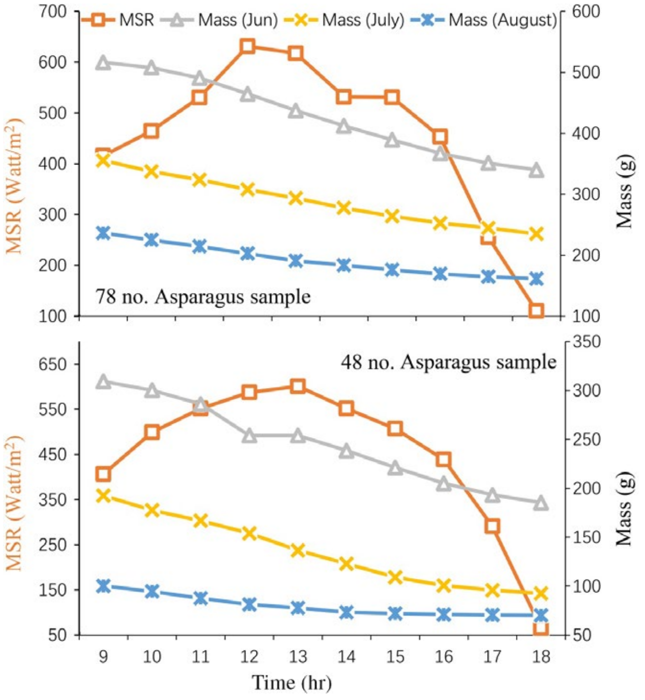

**FIGURE 3** The variation of solar irradiation and mass of the product with respect to time for 78 and 48 no. of asparagus samples; MSR shows the mean solar irradiance in watt/$m^2$ for the 3 months, *that is*, Jun, July, and August; Mass (Jun, July, and August) is the mass of the product of both 78 and 48 No. samples of asparagus

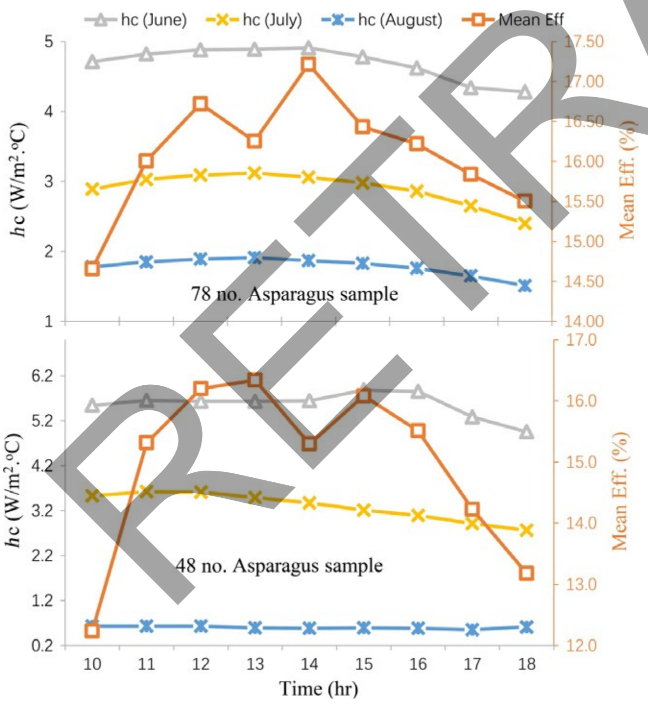

**FIGURE 4** The variations in convective heat transfer coefficients and efficiency with respect to time for 78 and 48 no. of asparagus samples; Mean Eff is the mean efficiency in (%) for all the 3 months; hc is the convective heat transfer coefficients in W/$m^2$ °C for the 78 and 48 No. of samples dried in the 3 months, *that is*, June, July, and August

$$Nu = \frac{h_c X}{K_v} = C(GrPr)^n \tag{5}$$

$$Q_e = 0.016 h_c [P(T_c) - \gamma P(T_e)] \tag{6}$$

$$Q_e = 0.016 \frac{K_v}{X} C(GrPr)^n [P(T_c) - \gamma P(T_e)] \tag{7}$$

$$m_{ev} = \frac{Q_e}{\gamma}(A_t t) = 0.016 \frac{K_v}{X} C(GrPr)^n [P(T_c) - \gamma P(T_e)](A_t t) \tag{8}$$

Let

$$0.016 \frac{K_v}{X\gamma}[P(T_c) - \gamma P(T_e)] = Z \quad \frac{m_{ev}}{Z} = C(GrPr)^n \tag{9}$$

Applying log to equation (9) on both side and we get equation (10),

$$\ln\left(\frac{m_{ev}}{Z}\right) = \ln C + n \ln(GrPr) \tag{10}$$

Similarly, applying the linear equation $y = mx + c$ on equation (10) and then we get

$$\ln\left(\frac{m_{ev}}{Z}\right), m = n, \text{ and } x = \ln(GrPr), c = \ln C \tag{11}$$

Values of "*m*" and "*C*" are obtained with the using of simple linear regression methods with the following formula.

$$m = \frac{N\sum X_0 Y - \sum X_0 \sum Y}{N\sum X_0^2 - \left(\sum X_0\right)^2} \tag{12}$$

And

$$c = \frac{\sum X_0^2 \sum Y - \sum X_0 \sum X_0 Y}{N\sum X_0^2 - \left(\sum X_0\right)^2} \tag{13}$$

The following relations were used for the determination of physical properties of humid air (Maskan et al., 2002; Misha, Mat, Ruslan, Salleh, & Sopian, 2015; Shanmugasundaram & Janarthanan, 2013).

$$C_v = 999.2 + 0.142 T_i + 1.101 \times 10^{-4} T_i^2 - 6.7581 \times 10^{-8} T_i^3 \tag{14}$$

$$K_v = 0.0244 + 0.7673 \times 10^{-4} T_i \tag{15}$$

$$K_v = \frac{353.44}{T_i + 273.15} \tag{16}$$

$$\mu_v = 1.718 \times 10^{-5} T_i + 4.620 \times 10^{-8} T_i \tag{17}$$

$$P(T) = \exp\left[25.317 - \frac{5144}{T_i + 273.15}\right] \text{ and } T_i = (T_c + T_e)/2 \tag{18}$$

The total heat of the collector outlet can be determined from the equation 19.

$$Q_0 = V_0 \times A_{0,c} \times \rho_v \times \left(T_{0,c} - T_{i,c}\right) \times c_v \tag{19}$$

The total amount of heat received by the solar flat-plate collector is given by equation (20).

$$Q_i = I \times A_c \tag{20}$$

The efficiency of solar flat-plate collector can be determined by dividing equations (19) and (20).

$$\eta_c = \frac{Q_0}{Q_i} \quad (21)$$

### 2.3.1 | Experimental errors

The experimental errors were evaluated in terms of percentage of uncertainty using equation (21) for the mass of moisture evaporated during drying of Asparagus samples (Tesfamariam et al., 2015; Tiago & Maria, 2014).

$$\%\,\text{uncertainity} = \left(\frac{U}{\text{mean of total observations}}\right) \times 100 \quad (22)$$

Where

$$U = \frac{\sqrt{\delta_1^2 + \delta_2^2 + \ldots + \delta_N^2}}{N_0} \quad (23)$$

## 3 | RESULTS AND DISCUSSION

The hand-peeled cylindrically shaped (diameter 1.7 cm, length 3 cm) different masses of Asparagus samples were dried under natural convection mode. Drying tests of different mass, *that is*, 78 and 48 no. of Asparagus samples were run using two different rectangular trays. Collector efficiency, moisture removing rate (%, dry basis), and convective heat transfer coefficients for 78 and 48 no. of Asparagus samples were evaluated as given in Table 3. The data given in table show the moisture removing rate, indoor and outdoor collector temperature, product and product surrounding temperature, and ambient temperature during the experiment. The data of solar irradiation and mass of product at drying time of 1-h interval under natural convection indirect solar drying for the month of June, July, and August 2016, of drying of 78 and 48 numbers of Asparagus samples are shown in Figure 3. The convective heat transfer coefficients and efficiency of the collector of drying of 78 and 48 numbers of Asparagus samples for the consecutive months of the year, 2016. It has been observed that the collector efficiency increases from morning to noon and decreases from noon to evening due to increasing and decreasing trend of solar irradiation in a day.

The maximum collector efficiency was reported between 12:00 and 14:00 as solar irradiation intensity was observed higher during the same time interval. So, the collector efficiency is observed to be a strong function of solar irradiation data (Fahim, Kang, et al., 2016; Gang et al., 2010; Kalogirou, 2009). The results of the study were in agreement with the findings by Jayashree et al., 2014; Karna & Koo, 2017; Kong et al., 2013. Similarly, the researchers (Kumar, 2014; Maskan et al., 2002; Norm, 2003) reported results were similar with our results; they studied that efficiency of the collector increased with increasing of solar irradiance.

The Table 3 shows the moisture removing rate is observed to be dependent on the total moisture present in the product mass, and hence, it has been observed that the moisture removing rate increases with increase in ginger samples mass and decreases significantly with the progression of drying days (El-Shobaki, El-Bahay, Esmail, Abd El-Megeid, & Esmail, 2010; Gang et al., 2010; Jamil, Osama, & Ahmed, 2014). However, the moisture removing rate is also dependent on the ease of heat transfer. Forced convection drying system has been reported to be best suitable for faster drying as the value of the coefficient of convective heat transfer associated with them is more than the natural convection drying (Azharul Karim & Hawlader, 2005; Deshmukh et al., 2013, 2014). From Figure 4, it has been observed that the values of convective heat transfer coefficient decrease with the progression of drying months, *that is*, June, July, and August 2016. This decrease in convective heat transfer coefficient value is due to a continuous reduction in moisture removal rate from the month of June to the next month of drying. The researchers (El-Shobaki et al., 2010; Fahim, Mansoor, et al., 2016; Meng et al., 2013; Vijaya, Iniyan, & Ranko, 2012) reported results were in agreement with our results of the study. The values of convective heat transfer coefficient have been observed to be dependent on the mass of fresh asparagus samples and decrease with increase in mass of the asparagus samples. So, it has been reported that the drying kinetics of asparagus is highly dependent on the mass is taken into consideration. The researchers (Jayashree et al., 2014; Karna & Koo, 2017; Kumar et al., 2011; Ünal, Alpsoy, & Ayhan, 2013) reported results were in closer agreements with our results of the study. They studied that convective heat transfer has been decreased with the increasing mass of the samples dried.

## 4 | CONCLUSIONS

The research reported in this study includes the evaluation of convective heat transfer coefficient, moisture removing rate, and collector efficiency for the different mass of asparagus samples under natural convection indirect solar drying mode. For analyzing the data with the help of linear regression method, we used Nusselt number expression. The following observations and conclusions have been made:

- Convective heat transfer coefficient was reported to vary from 1.78 to 4.74 W/m$^2$°C for 78 numbers asparagus samples, while 0.59 to 5.42 W/m$^2$°C noted for 48 numbers of asparagus samples.
- Convective heat transfer coefficients for both masses of asparagus samples decrease significantly with increase in the mass of asparagus samples.
- Moisture removing rate on a dry basis was observed to be increased with increase in asparagus samples mass and decreases significantly with the progression of drying months.
- Average collector efficiency during the drying process was observed to vary from 14.97 to 16.14%.
- Modified Henderson and Pabis were reported to be best suited for describing the drying behavior for both masses of asparagus samples.
- The experimental errors were evaluated in terms of percent uncertainty ranging from 29.19% to 46.25%.

RETRACTED

RETRACTED

## 5 | RECOMMENDATIONS

- The experimental errors occurred during the drying process further reduced using certain countermeasures such as sophisticated monitoring devices, design accuracy.
- The collector efficiency can be further improved using high conductive absorber material.
- The overall system efficiency can also be enhanced using phase change materials.
- The computer-based simulation tool was also an important method to study the design optimization and scalability of the system. The present research work could be considered for the optimum design of a solar dryer for quality drying of various products.

## ETHICAL STATEMENT

The submitting a paper do so on the understanding that the manuscript has been read and approved by all authors and agree to the submission of the manuscript to the Journal. All the authors have made an active contribution to the conception and design and/or analysis and interpretation of the data and/or the drafting of the manuscript, and all have critically reviewed its content and have approved the final version submitted for publication.

### ACKNOWLEDGMENTS

We thank Min Kang for providing laboratory facilities and extend our thanks to the China Scholarship Council and the College of Engineering, Nanjing Agricultural University, Nanjing, for supporting and providing research facilities for this study.

### CONFLICTS OF INTEREST

The authors declare no conflict of interest.

### ETHICAL REVIEW

This study does not involve any human or animal testing, and also this study was approved by the Institutional Review Board of Nanjing Agricultural University, China.

### INFORMED CONSENT

Written informed consent was obtained from all study participants.

### ORCID

*Fahim Ullah* http://orcid.org/0000-0002-3689-8743
